# Maintaining the Cartilage Phenotype of Late-Passage Chondrocytes Using Salidroside, TGF-β, and Sulfated Alginate for Cartilage Tissue Engineering Applications

**DOI:** 10.3390/ijms252413623

**Published:** 2024-12-19

**Authors:** Rita G. Diab, George Deeb, Rena Roda, Mia Karam, Marwa Faraj, Mohamad Harajli, Laila A. Damiati, Rami Mhanna

**Affiliations:** 1 Biomedical Engineering Program, Maroun Semaan Faculty of Engineering and Architecture, American University of Beirut, Beirut 1107 2020, Lebanon; rgd09@mail.aub.edu (R.G.D.); gdd01@mail.aub.edu (G.D.); rhr14@mail.aub.edu (R.R.); mpk05@mail.aub.edu (M.K.); mtf02@mail.aub.edu (M.F.); mh135@aub.edu.lb (M.H.); 2 Department of Biological Sciences, College of Science, University of Jeddah, Jeddah 21959, Saudi Arabia

**Keywords:** cartilage, chondrocytes, alginate sulfate, Salidroside, TGF-β, tissue engineering

## Abstract

The limited self-repair capacity of cartilage due to its avascular and aneural nature leads to minimal regenerative ability. Autologous chondrocyte transplantation (ACT) is a popular treatment for cartilage defects but faces challenges due to chondrocyte dedifferentiation in later passages, which results in undesirable fibroblastic phenotypes. A promising treatment for cartilage injuries and diseases involves tissue engineering using cells (e.g., chondrocytes), scaffolds (e.g., Alginate Sulfate (AlgSulf)), and biochemical signals (e.g., Salidroside and TGF-β). This study focuses on investigating the effects of AlgSulf scaffolds with varying degrees of sulfation, Salidroside, and TGF-β on the proliferation, viability, and phenotype maintenance of chondrocytes. The findings demonstrate that AlgSulf films with a degree of sulfation (DS) = 2, treated with a combination of Salidroside and TGF-β, significantly enhanced chondrocyte proliferation (*p* < 0.001 and *p* < 0.0001 in P2 and P4, respectively), preserved round cell morphology, and maintained cartilage-specific gene expression (Col2, Aggrecans, and SOX9) while downregulating fibroblastic markers (Col1, MMP13, IL-1β, and IL-6). Our findings suggest the potential of this combination for enhancing cartilage regeneration in tissue engineering applications.

## 1. Introduction

Osteoarthritis (OA) affects over 600 million people globally, with a higher rate of occurrence among women compared to men [1]. Women are more prone to the onset and progression of OA due to the decrease in sex hormone levels during menopause [2]. Furthermore, in contrast to male patients with OA, female patients are more likely to suffer from joint inflammation, thinner articular cartilage, greater clinical pain, and more severe joint movement problems [3]. Additionally, knee injuries are an important risk factor for developing knee OA. Post-traumatic OA can occur in previously injured joints. Research has shown that injured joints are five times more prone to developing OA than unharmed joints [4].

Cartilage can be divided into four zones: superficial, middle, deep, and calcified zones. Autologous chondrocyte implantation (ACI) [5] and matrix-associated chondrocyte implantation (MACI) [6] are the most common strategies for cartilage tissue engineering. These methods rely on multiplying harvested autologous chondrocytes on a 2D substrate without stimulation. The chondrocytes are then delivered with or without a matrix to injured sites. Due to monolayer expansion, chondrocytes used with these techniques have been shown to lose their cartilage phenotype and zonal characteristics [7]. In addition, the need for two surgeries (harvesting and implanting) causes further disadvantages, with potential complications, such as implant infections, which can, in some cases, be life-threatening. Currently, there are no efficient disease-modifying drugs, and the available surgical procedures are not able to fully restore the initial healthy state of the damaged articular cartilage. While ACI and MACI are promising, they have several limitations, essentially due to the limited number of chondrocytes available for transplantation, as well as the dedifferentiation of these cells upon passaging [8]. The synthesis of biomimetic sulfated polysaccharides has been utilized as a reliable model for cartilage engineering. Therefore, alginates have been considered biocompatible polysaccharides that could be sulfated and then applied in therapeutic procedures [9]. Salidroside [2-(4-hydroxyphenyl) ethyl beta-D-glucopyranoside] is a phenylalanine glycoside extracted from a plant known as Rhodiola rosea. Studies have found that Salidroside has pharmacological effects including boosting immunity, being hepatoprotective, having anti-cancerous and anti-oxidative properties, and eliminating melancholy [10]. Research has demonstrated that Salidroside can play an important anti-inflammatory role, which may help delay OA development by inhibiting the PI3K/AKT pathway, thus protecting chondrocytes [11]. Moreover, Salidroside was found to increase the expression of cartilage-specific genes such as Col2A1, Acan, and Sox9 while decreasing the expression of Col1A1, which is a dedifferentiation gene. The expression of Col2A1 is highly regulated by the Sox9 protein during the differentiation of chondrocytes [12]. Salidroside was also found to be able to significantly promote the secretion of glycosaminoglycans (GAGs) in chondrocytes and strengthen the extracellular matrix (ECM) network, hence protecting chondrocytes’ phenotype and enhancing their proliferation [1]. While the TGF-β/Smad3 signal has an essential function in ECM deposition and proliferation and the phenotype maintenance of chondrocytes, TGF-β inhibits the rate of maturation of chondrocytes. Smad2 and Smad3 are translocated to the nucleus in response to the TGF-β signal [13]. Salidroside was found to promote a surge in the levels of TGF-β and Smad3, showcasing the protective function that Salidroside exerts on chondrocytes [1]. A study by Zhang and Zhao illustrated that Salidroside has a significant effect on IL-1β-stimulated OA chondrocytes. It prevents IL-1β-induced NF-κB activation, and reduces nitric oxide, prostaglandin E-2, and matrix metalloproteinase production, while suppressing the expression of the inflammatory enzymes iNOS and COX-2, highlighting its potential as a therapeutic agent for OA [14]. An in vivo study by Gao et al. showed that Salidroside could significantly enhance the proliferation of chondrocytes in OA rats, upregulate the levels of Collagen II and Aggrecan, and downregulate the level of MMP-13. It also could reduce the number of CD4^+^IL-17^+^ cells and decrease the levels of IL-17, IKBα, and p65, while increasing the number of CD4^+^IL-10^+^ cells and the level of IL-10 [15]. Another study by Sun et al. illustrated that Salidroside promotes chondrocyte proliferation, enhances ECM synthesis, and prevents dedifferentiation by downregulating Col1 expression. On the other hand, in vivo, it accelerates hyaline cartilage repair during ACI while samples without Salidroside mainly undergo fibrous cartilage repair [16].

Here, we hypothesize that chondrocyte dedifferentiation will be significantly inhibited in chondrocytes grown on Alginate Sulfate thin films loaded with TGF-β and treated with Salidroside. These advanced Alginate Sulfate films represent a novel approach to improving chondrocyte functionality and maintaining their phenotype. By preventing dedifferentiation, this combination has the potential to address the critical limitation of losing cartilage-specific characteristics during in vitro expansion, paving the way for improved cartilage regeneration in tissue engineering applications.

## 2. Results

### 2.1. Proliferation Assay for P2 Chondrocytes

**Figure 1:**
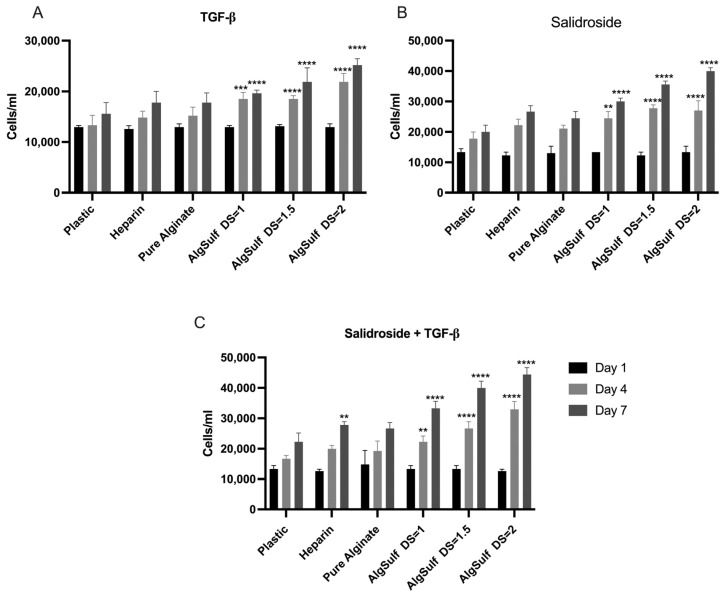
Proliferation assay for P2 chondrocytes subjected to TGF-β only (**A**), Salidroside only (**B**), and Salidroside and TGF-β (**C**). (**A**) In the presence of TGF-β only in the media, the cells cultured on Alginate Sulfate with a DS = 2 displayed the highest increase in chondrocyte numbers during the experiment, demonstrating that a higher DS offers the most suitable medium for cell growth on both day 4 and day 7. (**B**) The cells cultured in the presence of Salidroside only on Alginate Sulfate with a DS = 2 illustrated a higher proliferation rate on days 4 and 7 compared to the negative control (on plastic). (**C**) The presence of both TGF-β and Salidroside in the media displayed a significantly higher proliferation rate on days 4 and 7. Data are presented as mean ± SD. Statistical analysis was performed using two-way ANOVA and post hoc Tukey’s tests. Statistical significances are denoted as ** = *p* < 0.01; *** = *p* < 0.001; **** = *p* < 0.0001; *n* = 3.

For P2 chondrocytes grown on plastic in the presence of TGF-β only (Figure 1A), the cells first exhibited a gradual increase in numbers, reaching a plateau, indicating limited proliferation potential. In contrast, the cells cultured on Heparin showed a steady and significant surge in numbers during the experiment. When cultured on Alginate Sulfate with DS = 1 in the presence of TGF-β, P2 chondrocytes displayed significantly higher proliferation rates on day 4 (*p* = 0.001) and day 7 (*p* < 0.001) compared to the negative control. Similar trends were observed with DS = 1.5 and DS = 2, with the latter condition yielding the highest proliferation rate, demonstrating that a higher DS creates a more favorable environment for cell growth. Similarly, P2 chondrocytes grown in the presence of Salidroside only, on Alginate Sulfate with a DS = 1.5, showed a significantly higher proliferation rate on day 4 (*p* < 0.001) and day 7 (*p* < 0.001) when compared to the negative control. The same trend has been noticed with P2 chondrocytes grown on Alginate Sulfate with a DS = 2, which were also found to have a significant increase in proliferation rate on day 4 (*p* < 0.001) and day 7 (*p* < 0.001), in the presence of Salidroside only in the media (Figure 1B). The combination of Salidroside and TGF-β further enhanced proliferation rates across all DS conditions (DS = 1 (*p* = 0.003; day 4) (*p* < 0.001; day 7), DS = 1.5, (*p* < 0.001; day 4) (*p* < 0.001; day 7), and DS = 2 producing the highest cell density (*p* < 0.001; day 4) and (*p* < 0.001; day 7)). This combination demonstrated a synergistic effect, as the proliferation significantly exceeded the proliferation achieved by either Salidroside or TGF-β alone. Figure 1C displays a significant increase in chondrocyte proliferation. The increase in the number of cells proves that the combination of Salidroside and TGF-β promotes chondrocyte proliferation more efficiently than either treatment alone. 

### 2.2. Proliferation Assay for P4 Chondrocytes

**Figure 2:**
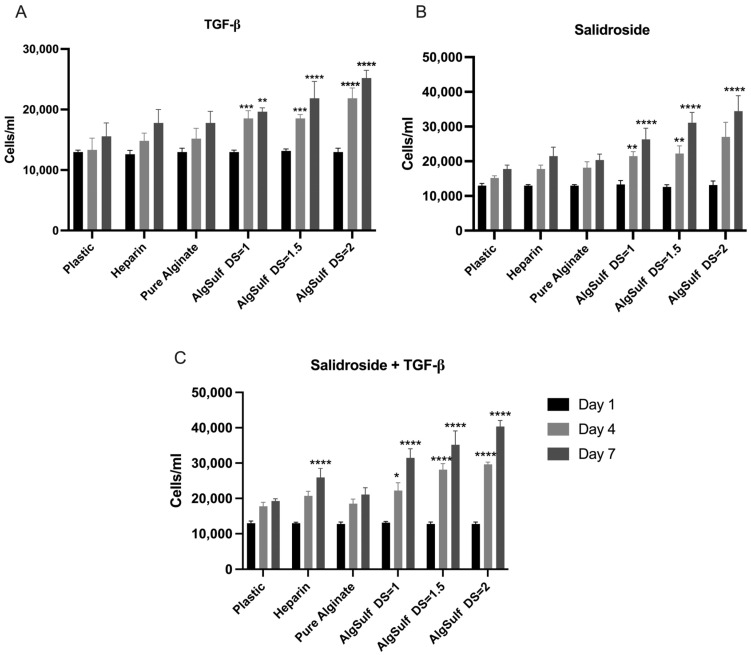
Proliferation assay for P4 chondrocytes subjected to TGF-β only (**A**), Salidroside only (**B**), and Salidroside and TGF-β (**C**). (**A**) The presence of TGF-β only in the media displayed the highest increase in chondrocyte amount during the experiment, demonstrating that a higher DS offers the most suitable medium for cell growth either on day 4 or 7. (**B**) Cells cultured in the presence of Salidroside only on Alginate Sulfate with a DS illustrated a higher proliferation rate on days 4 and 7 compared to the negative control. (**C**) The presence of both TGF-β and Salidroside in the media displayed a significantly higher proliferation rate on days 4 and 7. Data are presented as mean ± SD. Statistical analysis was performed using two-way ANOVA and post hoc Tukey’s tests. Statistical significances are denoted as * = *p* < 0.05; ** = *p* < 0.01; *** = *p* < 0.001; **** = *p* < 0.0001; *n* = 3.

P4 chondrocytes were treated with TGF-β under multiple conditions for seven days. The conditions tested consisted of negative control (on plastic), Heparin, Pure Alginate, and Alginate Sulfate with DS of 1, 1.5, and 2. The negative control and Heparin showed stable, minimal cell growth, with only a small number of cells over three days. On Pure Alginate, the number of chondrocytes moderately increased over time, with a slight enhancement of cell proliferation on day 4 (*p* = 0.3989) and day 7 (*p* = 0.2393) compared to the negative control. However, the most significant increases were recorded with Alginate Sulfate conditions. Specifically, Alginate Sulfate (DS = 1) showed a significant increase in chondrocyte numbers from day 1 to day 7 compared to the negative control (*p* = 0.0068). This impact was further amplified in Alginate Sulfate (DS = 1.5) (*p* < 0.0001). Finally, Alginate Sulfate (DS = 2) (*p* < 0.0001) displayed the highest proliferation rate, attaining the maximum cell count on day 7 (Figure 2A).

Figure 2B shows the P4 chondrocytes treated with Salidroside alone. The Heparin condition resulted in minimal, non-significant proliferation on day 4 (*p* = 0.4417) and on day 7 (*p* = 0.1469), while Pure Alginate showed a slight but non-significant increase in cell numbers on day 4 (*p* = 0.3184) and day 7 (*p* = 0.4408). Significant proliferation was observed with Alginate Sulfate conditions: DS = 1 showed a notable increase by day 4 (*p* = 0.0012) and day 7 (*p* < 0.0001), DS = 1.5 demonstrated even higher rates on the same days (*p* < 0.0001), and DS = 2 exhibited the highest proliferation, peaking on day 7 (*p* < 0.0001).

Figure 2C shows the P4 chondrocytes treated with a combination of Salidroside and TGF-β. Heparin resulted in low, stable cell numbers with no significant differences from the control (*p* < 0.0001; day 4) and (*p* < 0.0001; day 7). Pure Alginate showed moderate but non-significant proliferation (*p* = 0.9725; day 4) and (*p* = 0.5205; day 7). Significant increases were observed with Alginate Sulfate: DS = 1 showed notable growth by day 4 (*p* = 0.0101) and day 7 (*p* < 0.0001), further enhanced with DS = 1.5 (*p* < 0.0001), while DS = 2 exhibited the highest proliferation, peaking on day 7 (*p* < 0.0001).

### 2.3. Cell Viability Findings

**Figure 3:**
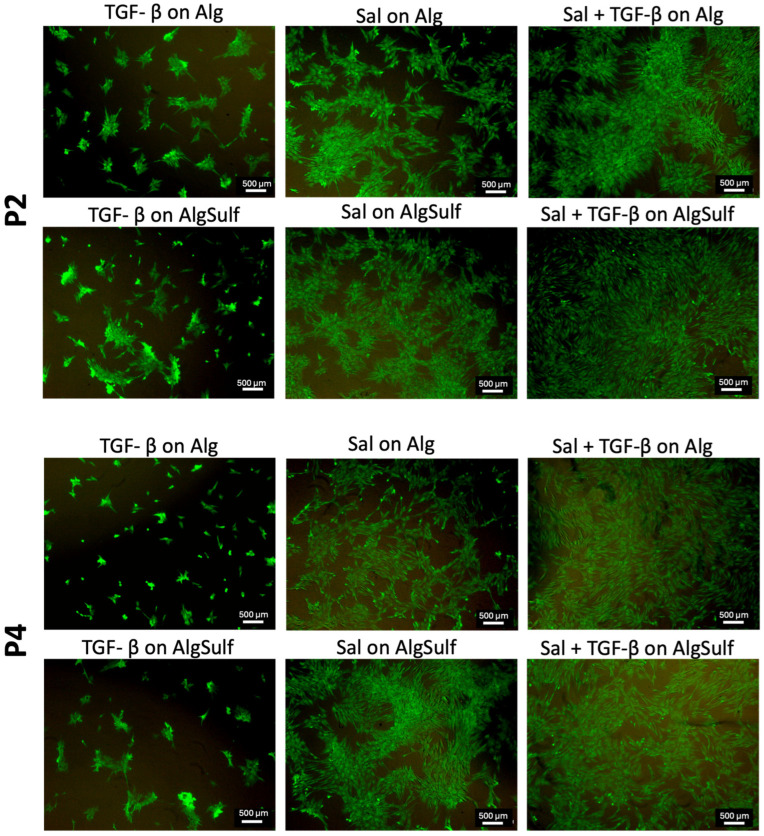
Live/Dead assay of P2 (**Top**) and P4 (**Bottom**) chondrocytes under various conditions. Treatments that include Salidroside, particularly when combined with TGF-β, consistently lead to higher cell viability and more enhanced protection of the chondrocyte phenotype. Alginate sulfate (DS = 2.0) optimizes the effects of Salidroside and TGF-β. The combination of Salidroside and TGF-β results in the highest cell viability and protection of chondrocytic phenotype. Scale bar = 500 μm.

After 7 days in culture, Live/Dead staining was used to assess cell viability and morphology. Figure 3 (Top) shows the P2 chondrocytes treated with Salidroside on Pure Alginate, exhibiting high green fluorescence, indicating high viability and maintaining a rounded or polygonal shape typical of healthy chondrocytes. When cultured on Alginate Aulfate (DS = 2.0) with Salidroside, the cells displayed even higher viability and a uniformly dispersed chondrocytic phenotype, suggesting enhanced protection and viability. The combination of Salidroside and TGF-β on Alginate Sulfate (DS = 2.0) resulted in the highest cell density and viability, with well-defined chondrocytic morphology, highlighting an optimal environment for maintaining the chondrocyte phenotype. In contrast, TGF-β treatment on Pure Alginate resulted in lower cell density and fibroblastic morphology, indicating dedifferentiation. TGF-β on Alginate Sulfate (DS = 2.0) improved viability slightly but maintained fibroblastic morphology compared to conditions containing Salidroside.

Figure 3 (Bottom) illustrates the P4 chondrocytes treated with Salidroside on Pure Alginate, showing partial maintenance of the chondrocytic phenotype. When cultured on Alginate Sulfate (DS = 2.0) with Salidroside, cell viability and the chondrocytic phenotype were notably improved. Salidroside combined with TGF-β on Pure Alginate resulted in high viability and chondrocytic morphology. However, TGF-β on Pure Alginate or Alginate Sulfate (DS = 2.0) resulted in lower viability and a mix of fibroblastic and chondrocytic morphologies, highlighting the limitations of TGF-β treatment alone.

### 2.4. Gene Expression

The expression and synthesis of type II collagen, type I collagen, SOX9, RUNX2, SZP, MMP13, and Aggrecan were assessed by qRT-PCR, for both P2 and P4 chondrocytes after 14 and 21 days.

**Figure 4:**
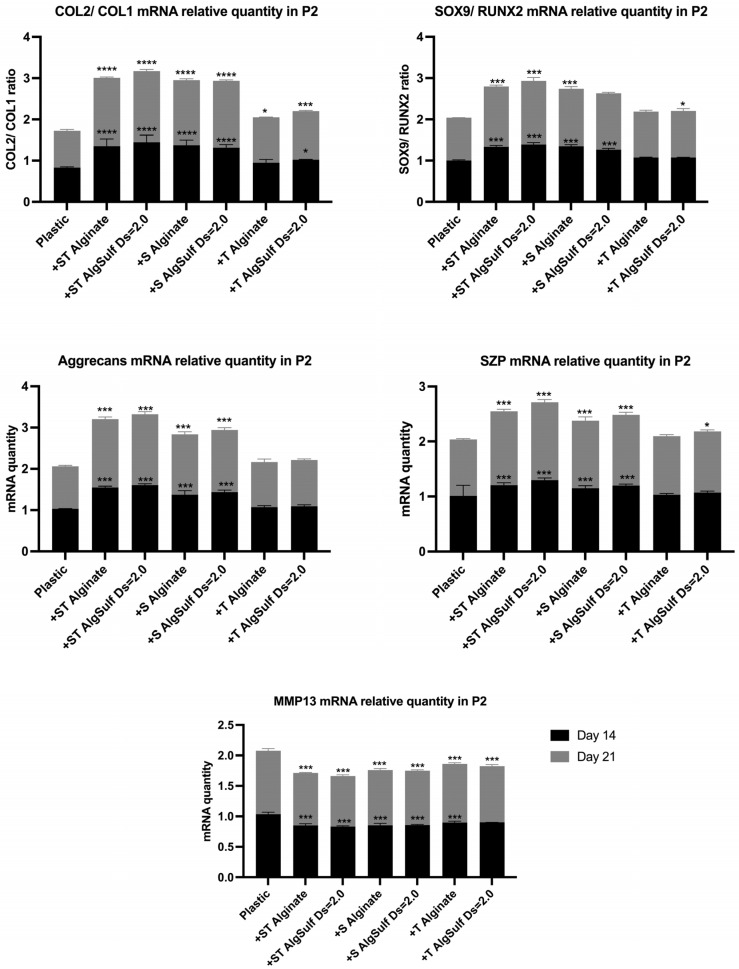
Col2/Col1, SOX9/RUNX2, Aggrecan, SZP, and MMP13 mRNA relative quantity in P2 chondrocytes. Cells were cultured on plastic (negative control), on Pure Alginate in the presence of Salidroside and TGF-β (+ST Pure Alginate), on Alginate Sulfate of DS = 2 in the presence of Salidroside and TGF-β (+ST Alginate Sulfate DS = 2.0), on Pure Alginate in the presence of Salidroside only (+S Pure Alginate), on Alginate Sulfate of DS 2 in the presence of Salidroside only (+S Alginate Sulfate DS = 2.0), on Pure Alginate in the presence of TGF-β only (+T Pure Alginate), and on Alginate Sulfate of DS 2 in the presence of TGF-β only (+T Alginate Sulfate DS = 2.0), on days 14 and 21. (The combination of Salidroside and TGF-β, with Alginate Sulfate of DS = 2.0, led to the highest Col2/Col1 ratio, SOX9/RUNX2 ratio, and SZP and Aggrecan mRNA levels, suggesting the best condition to prevent chondrogenic dedifferentiation. In the presence of Salidroside alone, or combined with TGF-β, MMP13 mRNA levels were significantly reduced, especially on alginate sulfate.) Results are shown as a ratio to negative control. Data are presented as mean ± SD. Statistical analysis was performed using two-way ANOVA and post hoc Tukey’s tests. Statistical significances are denoted as * = *p* < 0.05; *** = *p* < 0.001; **** = *p* < 0.0001; *n* = 3.

**Figure 5:**
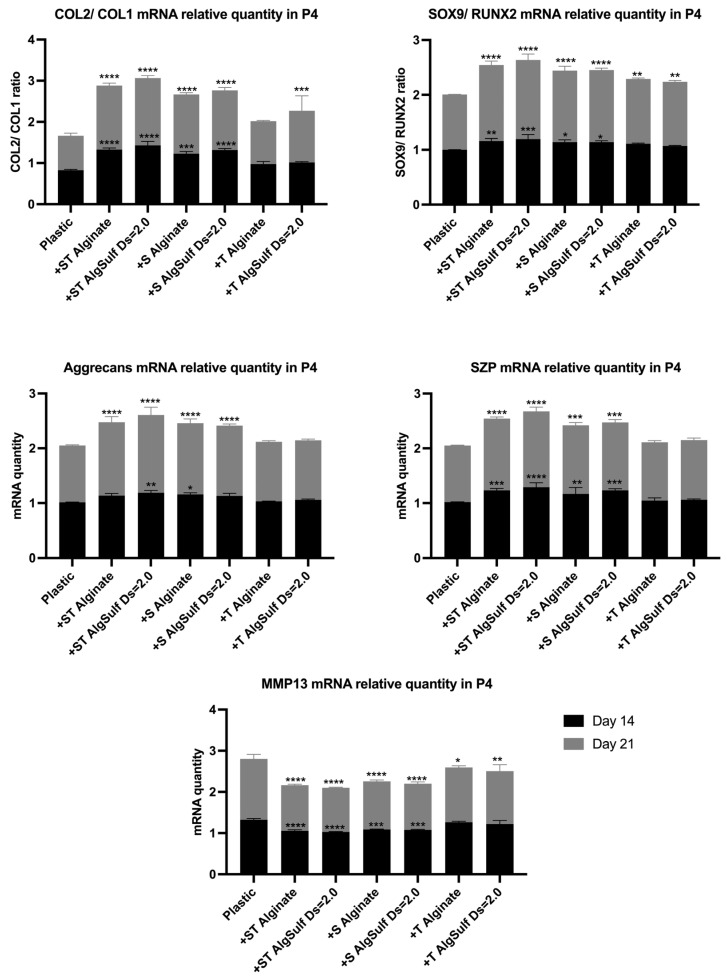
Col2/Col1, SOX9/RUNX2, Aggrecan, SZP, and MMP13 mRNA relative quantity in P4 chondrocytes. Cells were cultured on plastic (negative control), on Pure Alginate in the presence of Salidroside and TGF-β (+ST Pure Alginate), on Alginate Sulfate of DS 2 in the presence of Salidroside and TGF-β (+ST Alginate Sulfate DS = 2.0), on Pure Alginate in the presence of Salidroside only (+S Pure Alginate), on Alginate Sulfate of DS = 2 in the presence of Salidroside only (S Alginate Sulfate D = 2.0), on Pure Alginate in the presence of TGF-β only (+T Pure Alginate), and on Alginate Sulfate of DS = 2.0 in the presence of TGF-β only (+T Alginate Sulfate DS = 2.0), on days 14 and 21. (The combination of Salidroside and TGF-β, with Alginate Sulfate DS = 2.0, resulted in the highest Col2/Col1 ratio, SOX9/RUNX2 ratio, SZP, and Aggrecan mRNA levels. highlighting the role of this treatment in providing a suitable chondrogenic environment for P4 chondrocytes. This treatment was also the best at downgrading the levels of MMP13 mRNA levels, especially when P4 was grown on alginate sulfate.) Results are shown as a ratio to negative control. Data are presented as mean ± SD. Statistical analysis was performed using two-way ANOVA and post hoc Tukey’s tests. Statistical significances are denoted as * = *p* < 0.05; ** = *p* < 0.01; *** = *p* < 0.001; **** = *p* < 0.0001; *n* = 3.

The Salidroside and TGF-β combination, particularly on alginate sulfate, led to enhanced cell–matrix interactions, an optimized retention of growth factors, and the suitable expression of key chondrogenic markers such as Col2, Aggrecans, and SOX9 while reducing dedifferentiation markers such as Col1 and inhibiting the catabolic activity revealed by decreased MMP13 expression (Figure 4 and Figure 5).

To assess whether the mechanism of action of Alginate Sulfate is mediated through interleukin (IL) signaling, the expression of IL-1β and IL-6 was measured, and the results indicated that Alginate Sulfate mediated its effects through IL signaling. The lowest levels of IL-1β and IL-6 expression were observed in chondrocytes cultured with Alginate Sulfate (DS = 2.0) (Supplementary Figure S1).

### 2.5. Protein Expression

**Figure 6:**
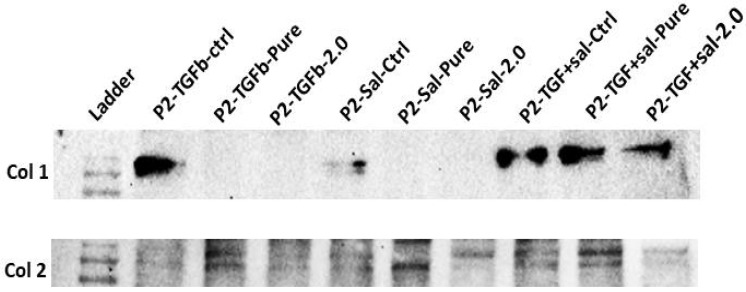
Western blot results of Col1 and Col2 expression for P2 chondrocytes under various treatment conditions.

The Western blot experiment accurately illustrated the expression profiles of Col1 and Col2 among various treatment conditions applied to P2 chondrocytes. The negative control condition showed a high level of Col1 expression and very low Col2 expression, indicating substantial dedifferentiation. In contrast, chondrocytes treated with TGF-β or Salidroside alone exhibited a reduction in Col1 expression and an increase in Col2 expression, particularly when these factors were combined with Alginate Sulfate scaffolds. Notably, the Alginate Sulfate group displayed the lowest levels of Col1 and the highest levels of Col2 among all conditions, highlighting the most significant preservation of the chondrocyte phenotype (Figure 6).

## 3. Discussion

In this study, the proliferation of Passage 2 (P2) and Passage 4 (P4) chondrocytes was evaluated under various conditions: on plastic (negative control), on Heparin, on Pure Alginate, and on Alginate Sulfate with degrees of sulfation of 1, 1.5, and 2, all in the presence of TGF-β, Salidroside, and the combination of the two. Alginate sulfate has been shown to mimic the ECM due to its sulfation, which provides an environment conducive to chondrocyte proliferation and matrix synthesis [17]. For both P2 and P4 chondrocytes, the optimal proliferation was obtained when cells were grown on Alginate Sulfate of DS = 2.0, in the presence of Salidroside and TGF-β. This finding aligns with previous studies showing the ability of Alginate Sulfate to provide a suitable environment that both enhances chondrocyte proliferation and maintains the chondrogenic phenotype [18,19]. The ability of Salidroside to maintain chondrocyte morphology and prevent dedifferentiation, as highlighted in this study, aligns with its known anti-apoptotic and anti-inflammatory properties that assist in protecting cells under stress and preserving their function during passaging [20]. TGF-β is a key regulator of chondrocyte differentiation, but its efficacy is improved when combined with other factors such as alginate sulfate, which provides a better matrix for cellular attachment and growth [21]. Therefore, both Salidroside and TGF-β promote chondrocyte proliferation [16], which aligns with the results obtained from this study.

The Live/Dead assay performed on both P2 and P4 chondrocytes showed that the highest cell viability occurred in the presence of Salidroside and TGF-β when cells were grown on Alginate Sulfate (DS = 2.0). In addition, in the presence of Salidroside, alone or when combined with TGF-β, the chondrocytes exhibited a round morphology, typical of healthy chondrocytes, showing that Salidroside alone can maintain cell viability and the chondrogenic phenotype efficiently. Conversely, when the chondrocytes were subjected to TGF-β on Pure Alginate, the cell density was lower compared to conditions containing Salidroside, and the chondrocytes displayed fibroblastic morphology, signaling a shift toward dedifferentiation. This observation aligns with previous experiments, which revealed Salidroside’s ability to maintain the chondrocyte phenotype and protect morphology during passaging, thereby preventing dedifferentiation [16].

The PCR analysis performed on both P2 and P4 chondrocytes showed that the combination of Salidroside and TGF-β, with Alginate Sulfate of DS = 2.0, led to the highest Col2/Col1 ratio, suggesting that it is the most efficient among all other tested conditions, preventing chondrogenic dedifferentiation. This treatment also led to the highest Aggrecan and SZP mRNA levels and the highest SOX9/RUNX2 ratios, highlighting its role in providing a suitable chondrogenic environment, promoting matrix synthesis, and optimizing the production of proteins crucial for cartilage function. Treatments that consisted of Salidroside alone, or combined with TGF-β, resulted in a reduction in MMP13 mRNA levels, especially when the cells were grown on alginate sulfate. This implies a reduction in matrix degradation, creating a more stable and protective environment for cartilage. Previous studies have also found that Salidroside can efficiently upregulate the levels of Col2 and Aggrecans and downregulate the level of MMP-13 and Col1 [15], which corresponds with the results obtained in our study. This finding is consistent with the results by Zhang and Zhao [14] and Sun et al. [16], who demonstrated that Salidroside can upregulate the levels of Col2 and Aggrecan, while downregulating the expression of MMP-13 and Col1. Furthermore, our findings suggest that the mechanism of action of Alginate Sulfate is mediated through ILs, particularly IL-1β and IL-6 expression, supporting the hypothesis that Alginate Sulfate exerts its effects through an IL-mediated pathway. This aligns with existing studies highlighting the critical role of ILs in inflammatory and chondrocyte regulatory processes [22,23].

The Western blot analysis of P2 chondrocytes demonstrated a consistent reduction in the expression of Col1 when transitioning from plastic to alginate and further to alginate sulfate, emphasizing the efficiency of these substrates in maintaining chondrocyte phenotype. Specifically, alginate sulfate, with its higher degree of sulfation, provides a more suitable microenvironment for chondrocytes compared to Pure Alginate, due to better ECM mimicry and enhanced cell–matrix interactions [24]. Furthermore, research has demonstrated that sulfated alginates possess potent anti-inflammatory and antioxidant properties. Specifically, they reduce the expression of pro-inflammatory markers such as Il-6 and CXCL8 in chondrocytes exposed to IL-1β, with their effectiveness correlating to the degree of sulfation [25]. A review by Kerschenmeyer et al. illustrated that various factors such as the presence of FGF-2 and different hypoxic conditions can influence the cellular response, which can enhance the repair mechanism by promoting chondrocytes survival and matrix synthesis [26]. However, further investigations are required to fully understand the underlying mechanism. Treatments with TGF-β alone and Salidroside alone, especially when combined with alginate sulfate, led to the reduction in Col1 expression and simultaneously increased the amount of Col2, consistent with the results obtained from the PCR experiment. This effect portrays the therapeutic potential of these conditions in not only limiting chondrocyte dedifferentiation but also promoting the expression of essential matrix components.

## 4. Materials and Methods

### 4.1. Chondrocyte Isolation

Chondrocytes were isolated from the knees of 6-month-old calves sourced from a local slaughterhouse, abiding by the established protocols [27]. Cartilage shavings were sliced using a sterile blade and were then incubated with 0.2% pronase in DMEM that contains 1% antibiotic-antimycotic for 2 h at 37 °C and 7% CO_2_ with mild stirring. After being digested by pronase, the tissue was washed three times using DMEM containing 1% antibiotic-antimycotic and then incubated for 6 h in 0.03% collagenase in DMEM supplemented with 1% antibiotic-antimycotic at 37 °C and 7% CO_2_ with gentle stirring. Cells were isolated from the digested matrix by filtering them through 100 μm and 40 μm cell strainers in sequences. Cell counting and viability evaluation were carried out using an automated cell counter (Countess™ Automated Cell Counter, Invitrogen AG, Basel, Switzerland), with viability surpassing 90% for all separations. The isolated cells were cultured at a density of 10,000 cells/cm^2^ in DMEM along with 1% antibiotic-antimycotic, 10% fetal bovine serum (FBS), and 50 μg/mL L-ascorbic acid. When the cells reached 80–90% confluency, they were removed by adding trypsin/EDTA and then seeded again at a density of 5000 cells/cm^2^ for later passages.

### 4.2. Cell Culture

Passage (P) P0 and P2 chondrocytes were seeded and expanded until P2 and P4, respectively, in T75 Flasks containing DMEM-F12 media supplemented with 10% FBS, 1% penicillin-streptomycin, and 50 μg/mL ascorbic acid (Sigma-Aldrich, Dorset, UK). Cells were harvested with trypsin/EDTA and then seeded on the prepared 2D films.

### 4.3. Two-Dimensional Film Build-Up and Cell Seeding

All materials were purchased from Sigma-Aldrich unless otherwise stated. Thin films of Alginate Sulfate were prepared using biotin–streptavidin bonding [28,29]. Briefly, 200 μL of 0.1 mg/mL biotinylated bovine serum albumin (bBSA) was added in each well of the 48-well plate and incubated at 4 °C overnight. After washing the plates with phosphate-buffer saline (PBS), 200 μL of 25 μg/mL streptavidin was added to each well of the plate and incubated for 2 h at room temperature. Next, and after washing the plates with PBS, biotinylated Alginate Sulfate with different sulfation degrees (0.0, 1.0, 1.5, and 2.0) was added for 1 h at RT, after which they were washed again with PBS. DMEM-F12 complete medium (10% FBS, 1% penicillin-streptomycin, and 50 µg/mL ascorbic acid) was added to the wells, and incubated for 1 h. Wells without coating served as negative controls and wells containing Heparin side-on and end-on served as positive controls for the alginate sulfate.

P2 and P4 chondrocytes were cultured on the thin films in 48-well plates at a seeding density of 50 × 10^3^ cells per well (this density was chosen to observe a measurable proliferation without the overcrowding of the cells), in various conditions, on different substrates: (1) Plastic (as negative control), (2) Heparin, (3) AlgSulf0.0, (4) AlgSulf1.0, (5) AlgSulf1.5, and (6) AlgSulf2.0. Chondrocytes were subjected to each of these conditions in three groups as follows: (1) Chondrocytes + TGF-β (10 ng/mL) + Salidroside (1.33 μM), (2) Chondrocytes + Salidroside (1.33 μM; this concentration was chosen as it demonstrated the best regulation in promoting cartilage-specific markers while inhibiting dedifferentiation markers [16]), and (3) Chondrocytes + TGF-β (10 ng/mL), to compare the effect of the combination of Salidroside and TGF-β, to that of each agent on its own, on the proliferation of P2 and P4 chondrocytes. P2 and P4 chondrocytes were incubated in a 37 °C 5% CO_2_ incubator.

### 4.4. Cell Viability

On day 7 of the treatment, the Live/Dead assay (Live/Dead^®^ viability assay, Life technologies, Paisley, UK) was performed to detect the cell’s viability and check for the toxicity of Salidroside. The cells were stained using 150 μL solution containing 0.1 μM Ethidium homodimer and 1 μM calcein AM. The cells were then incubated for 35 min, washed with PBS, and observed under a fluorescent microscope (Carl Zeiss, Baden-Württemberg, Germany).

### 4.5. qRT-PCR

The cells were washed with PBS and lysed with TRIzol. The lysate was transferred to Eppendorf tubes and stored at −20 °C. Later, chloroform was added and centrifuged at 12,000× *g* for 15 min at 4 °C, resulting in three phases: organic (proteins), interphase (DNA), and aqueous (RNA). The RNA-containing aqueous phase was collected, isopropanol was added, and the RNA was precipitated via centrifugation. The RNA pellet was washed twice with 75% ethanol, resuspended in RNAse-free water, and incubated at 60 °C. RNA concentration was measured using a Nanodrop, and reverse transcription (RT) was performed for qRT-PCR according to the manufacturer’s instructions (Qiagen, Valencia, CA, USA). The expression of type II collagen, type I collagen, SOX9, RUNX2, SZP, MMP13, and Aggrecans were assessed for both P2 and P4 chondrocytes. PCR was also performed for IL-6 and IL-1β to evaluate their expression in late-passage chondrocytes grown on alginate sulfate, aiming to test their inhibition properties. The conditions tested included normal oxygen tension (20%) with and without FGF-2, as well as hypoxic conditions (1% oxygen tension) with and without FGF-2. Hypoxia was used to mimic the native cartilage tissue environment, while FGF-2 was included to investigate its action under hypoxic conditions. The 2^−ΔΔCT^ method was used for the interpretation [30]. The genes investigated in this study and the primers used are listed in Table 1.

### 4.6. Western Blot

Cells were harvested using a cell scraper and were either processed directly or snap-frozen and then stored at −80 °C. Lysis was performed on ice for 15 min in RIPA buffer containing protease and phosphatase inhibitors, followed by centrifugation at 13,000 rpm for 10 min. The supernatant containing proteins was collected, and protein quantification was performed using the Bradford assay. Samples were stored at −20 °C if not used immediately. For the Western blot assay, a glass setup was assembled for gel casting. Resolving and stacking gels were prepared, and protein samples were boiled with 1X Laemmli buffer before loading into the gel. Electrophoresis was performed at 80 V until samples reached the resolving gel and the run continued at 140 V. Afterwards, proteins were transferred onto a nitrocellulose membrane in a sandwich setup with a transfer buffer at 30 V overnight in a cold room. Next, the membrane was stained with Ponceau red for 1 min and then washed with water to visualize transferred proteins. Blocking was performed, with either 5% milk or 3% BSA in phosphate-buffered saline-Tween 20 (PBS-T) (depending on the primary antibody used) for 1 h at RT. Incubation with primary antibodies against Collagen I (II-II6B) and Collagen II (8-3A5), both from the Developmental Studies Hybridoma Bank (DSHB), IA, was carried out overnight, followed by 3 rounds of washing with PBS-T for 10 min each. Next, the membrane was incubated with goat anti-mouse horseradish peroxidase (HRP) conjugated secondary antibody (Santa-Cruz Biotechnology, Dallas, TX, USA) at a concentration of 1:5000 in 3% BSA in PBS-T for 1 h at RT. After 3 rounds of washing as before, enhanced chemiluminescence (ECL) substrate (Bio-Rad, Richmond, CA, USA) was added, and the blot was then observed on the ChemiDoc imaging system (Bio-Rad, Richmond, CA, USA). For stripping, the membrane was incubated in stripping buffer for 20 min at RT on a shaker, and washed twice for 5 min each with PBS-T; the stripping buffer was prepared by dissolving 3.75 g glycine, 2.5 mL 10% SDS, and 2.5 mL Tween 20 in 250 mL autoclaved water, adjusting pH to 2.2 with HCl, and stored at 4 °C.

### 4.7. Statistical Analysis

Data were obtained from the samples and represented as the mean ± standard deviation (SD). Statistical assessment was carried out using two-way ANOVA and post hoc Tukey’s tests where *p*-values of less than 0.05 were considered significant. Statistical analysis was performed using Prism software (GraphPad version 10.2.3 (403), San Diego, CA, USA).

## 5. Conclusions

This work highlights the synergistic effect of Salidroside and TGF-β to significantly promote proliferation and viability, and prevent the chondrogenic dedifferentiation of late-passage chondrocytes cultured on Alginate Sulfate matrices. The findings obtained from the various assays shed light on Salidroside’s antioxidative and anti-inflammatory characteristics, along with TGF-β’s potent chondrogenic potential, which provides an optimal environment for maintaining the phenotype of chondrocytes and preventing their dedifferentiation. This combinatory treatment, particularly on an Alginate Sulfate matrix, leads to enhanced cell–matrix interactions, an optimized retention of growth factors, and the suitable expression of key chondrogenic markers such as Col2, Aggrecans, and SOX9 while reducing dedifferentiation markers such as Col1 and inhibiting the catabolic activity revealed by decreased MMP13 expression. Furthermore, the current findings may highlight the potential of AlgSulf in enhancing cartilage repair by mitigating pro-inflammatory responses, particularly under different oxygen tensions and in the presence of FGF-2. These results have promising implications for approaches involving cartilage tissue engineering to treat OA. These findings indicate that such efficient treatments can optimize chondrocyte performance and result in more efficient cartilage repair and regeneration techniques. Although the sulfation of alginate provided an enhanced environment for chondrocytes, the ECM of native cartilage is a complex structure composed of multiple components and specific biochemical cues. AlgSulf alone may not fully replicate this complexity. Future studies could explore incorporating additional ECM components, such as collagen or hyaluronic acid, into the scaffold to better mimic native cartilage ECM. Also, more work should delve into the molecular processes regulating the synergistic effects of this treatment to better refine and benefit from their potential in tissue engineering procedures.

## Figures and Tables

**Table 1:** Primers used for qRT-PCR.

Gene Name	Primer Sequence
*Col1*	F-CGAGGGCAACAGCAGATTCAC TTA R-GCA GGC GAG ATG GCT TGT TTG
*Col2*	F-GGC CAG CGT CCC CAA GAA R-AGCAGGCGC AGG AAG GTC AT
*SOX9*	F-ACG CGG CCC CAG GAG AAC R-CGGATG CACACGGGGAACTT
*RUNX2*	F-TTTTCAGACCCCAGGCAGTT R-TTGGAGAAGCGGCTCTCAGT
*Aggrecan*	F-AGTAGAGGACATCAGCGGGCTT R-CCGCTGATGTCCTCTACTCCAG
*SZP*	F-TTGCGCAATGGGACATTAGTT R-AGCTGGAGATGGTGGACTGAA
*MMP13*	F-CCTTGATGCCATTACCAGTCTCC R-AAACAGCTCCGCATCAACCTGC
*IL-1*β	F-ACCCCAAAGTCTACCCCAAG R-CCAGTTAGGGTACAGGACAGAC
*IL-6*	F-GTGCTCCTGGTGATGACTT R-GAAGTAGTCTGCCTGGGGTG

## Data Availability

The original contributions presented in the study are included in the article; further inquiries can be directed to the corresponding authors.

## Supplementary Materials

The following supporting information can be downloaded at: https://www.mdpi.com/article/10.3390/ijms252413623/s1.
