# Supplementary material for: Maintaining the Cartilage Phenotype of Late-Passage Chondrocytes Using Salidroside, TGF-β, and Sulfated Alginate for Cartilage Tissue Engineering Applications
Source: Int J Mol Sci. 2024 Dec 19;25(24):13623. doi: 10.3390/ijms252413623 (PMC11727720; doi:10.3390/ijms252413623)
Supplement: Supplementary file 1 [file ijms-25-13623-s001.zip › ijms-3322831-supplementary.pdf]

Supplementary data for:

Maintaining the Cartilage Phenotype of Late Passage Chondrocytes Using Salidroside, TGF-β and Sulfated Alginate for Cartilage Tissue Engineering Applications

Rita G. Diab[1], George Deeb[1], Rena Roda[1], Mia Karam[1], Marwa Faraj[1], Mohamad Harajli[1], Laila A. Damiati[2*], Rami Mhanna[1*]

[1] Biomedical Engineering Program, Maroun Semaan Faculty of Engineering and Architecture, American University of Beirut, Beirut 1107 2020, Lebanon, R.G.D rgd09@mail.aub.edu, G.D gdd01@mail.aub.edu, R.D rhr14@mail.aub.edu, M.K mpk05@mail.aub.edu, M.F mtf02@mail.aub.edu, M.H mh135@aub.edu.lb, R.M rm136@aub.edu.lb

[2] Department of Biological Sciences, College of Science, University of Jeddah, Jeddah 21959, Saudi Arabia

*Correspondence: R.M rm136@aub.edu.lb ; L.A.D ladamiati@uj.edu.sa

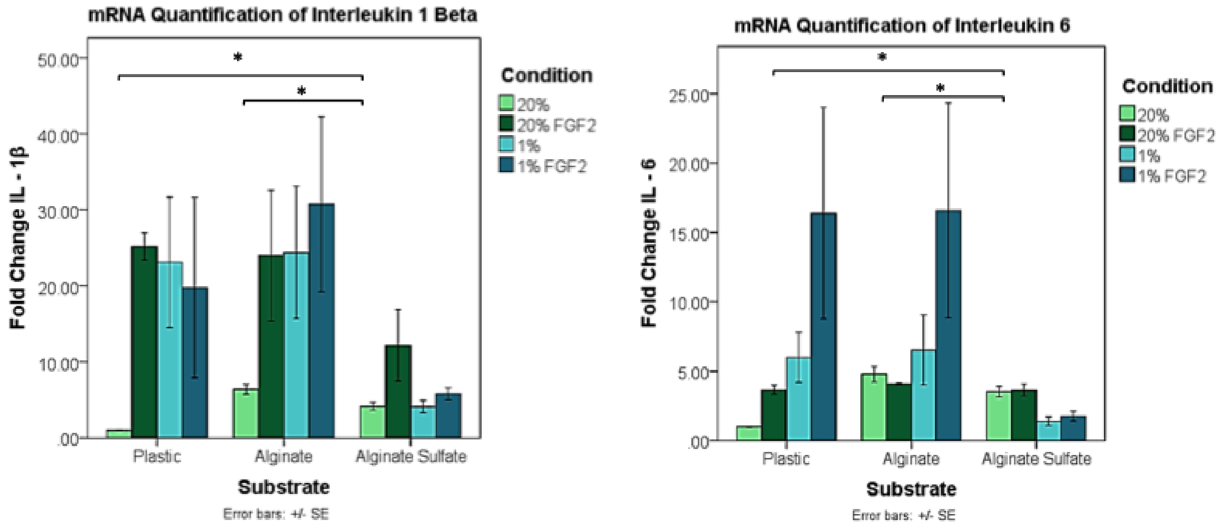

Supplementary Figure S1: Alginate sulfate reduces inflammatory cytokine expression in chondrocytes. IL-1ß and IL-6 levels were measured using quantitative PCR in chondrocytes cultured with alginate sulfate and compared to negative control (plastic). Data shows significant downregulation of IL-1ß and IL-6 in AlgSulf-treated (DS=2.0) samples when compared to alginate, supporting its role in modulating inflammation via IL-mediated pathways. The lack of significant differences when compared to plastic is likely due to minimal extracellular matrix formation on plastic, which inherently reduces inflammatory signaling. These findings suggest that AlgSulf enhances cartilage repair by mitigating pro-inflammatory responses. The conditions in the figure (20%, 20% FGF, 1%, and 1% FGF) refer to normal oxygen tension (control), normal oxygen tension with basic fibroblastic growth factor (FGF-2), 1% oxygen tension, and 1% oxygen tension with FGF-2, respectively. Results are presented as mean ± SD, with statistical significance indicated *$p<0.05$.
